# 기술수용모델 2를 중심으로 한국 간호대학생의 메타버스 기반 블랜디드러닝 간호해부학 수업 수용의도에 영향을 미치는 요인: 횡단적 연구

**DOI:** 10.7586/jkbns.25.061

**Published:** 2025-11-26

**Authors:** Mi Yang Jeon, Eunju Heo, Chi Yang Yoon, Won Min Jeong, Hyeon Cheol Jeong

**Affiliations:** 1 College of Nursing, Sustainable Health Research Institute, Gyeongsang National University, Jinju, Korea; 1 경상국립대학교 간호대학•지속가능건강연구소; 2 Department of Nursing, Changwon National University, Changwon, Korea; 2 국립창원대학교 간호학과; 3 College of Nursing, Gyeongsang National University, Jinju, Korea; 3 경상국립대학교 간호대학; 4 College of Nursing, Sahmyook University, Seoul, Korea; 4 삼육대학교 간호대학

**Keywords:** Students, nursing, Intention, Anatomy, 간호대학생, 의도, 해부학

## Abstract

**Purpose:**

This study aimed to identify the factors influencing nursing students’ intention to use metaverse-based blended learning in nursing anatomy classes by applying the technology acceptance model 2 (TAM 2).

**Methods:**

This descriptive survey study included 155 students from a nursing college in Seoul. TAM 2 comprised 25 items measured on a 5-point Likert scale, encompassing subjective norm, image, job relevance, output quality, result demonstrability, perceived ease of use, perceived usefulness, and intention to use. Hierarchical regression analysis was performed to identify factors affecting students’ intention to use metaverse-based learning.

**Results:**

In regression model 1, which included general and metaverse education-related characteristics, the factors significantly influencing the intention to use were the perceived necessity of metaverse education (β = .59, *p* < .001) and having more than three semesters of metaverse experience (β = −.22, *p* = .010), explaining 41.0% of the variance. When TAM 2 variables were added (model 2), significant predictors included perceived necessity of metaverse education (β = .26, *p* = .004), perceived ease of use (β = .33, *p* < .001), and perceived usefulness (β = .48, *p* < .001), explaining 76.0% of the variance.

**Conclusion:**

To promote effective use of the metaverse platform in nursing education, strategies that enhance students’ perceived usefulness and ease of use are essential. Educational interventions should focus on improving familiarity with metaverse technologies to facilitate their integration into nursing curricula.

## 서론

### 1. 연구의 필요성

간호학에서 간호해부학은 간호대학생의 전공교과목 이해도를 높이기 위해 필수적인 인체의 구조와 기능을 교육하는 전공기초교과목이다[1]. 간호학과에서는 해부학을 전공교과목 전 단계인 1, 2학년에게 교육하고 있어 간호대학생들은 해부학을 익숙하지 않은 암기해야 할 의학용어가 많은 힘든 교과목으로 인식하고, 시험을 위한 단순 암기 위주로 학습하는 경우가 많다[2]. 이에 간호학과에서는 간호대학생의 인체 구조에 대한 이해를 높이기 위해 카데바를 이용한 해부학실습 교육을 병행하고 있다. 해부학실습은 단순히 해부학적 지식 습득을 넘어, 의료인의 인간 중심적 가치와 정체성을 형성하는 중요한 과정으로, 인간의 존엄성과 생명의 가치에 대해 깊이 성찰할 수 있는 기회를 제공한다[3]. 그러나 최근 의과대학에서도 시신 확보의 어려움, 기초의학 교수진 및 지원인력의 부족, 실습환경의 미비 등 여러 구조적인 문제로 해부학실습을 학습할 수 있는 기회를 충분히 제공하고 있지 못하다[4]. 더욱이 간호학과는 단독으로 해부학실습을 할 수 없을 뿐 아니라 의과대학이 있는 대학의 간호대학생도 해부학실습을 경험하기에는 기회가 제한적이다[5]. 이에 간호학과에서는 해부학 수업이나 해부학실습에서 동영상, 3차원(three dimensions) 영상, 가상현실(virtual reality, VR)/증강현실(augmented reality, AR)과 같은 4차 산업기술을 적용한 교수학습방법을 적용하고 있다[6]. 그러나 이러한 교수학습방법을 활용한 교육은 일방향적으로 이루어지기 때문에 학습자-교수자간 상호작용이 제한적이며, 학생들의 학업성취를 확인하는데 제한점이 있다. 그러므로 간호대학생의 해부학에 대한 흥미와 이해를 높이기 위해서는 교수자-학습자 또는 학습자간 상호작용을 하면서 학습할 수 있는 교수학습방법이 필요하다.

최근 4차 산업혁명의 가속화로 현실과 가상세계의 경계를 넘어 새로운 미래공간으로의 변화가 가능해지면서 메타버스(metaverse)에 대한 관심이 증가하고 있다[7]. 메타버스는 가상, 초월을 뜻하는 메타(meta)와 세계, 우주를 의미하는 유니버스(universe)의 합성어로, 가상 디지털 세계를 뜻한다[8]. 메타버스 기반 교육에서는 학습자가 교수자 또는 동료들과 상호작용할 수 있어 기존의 일방향적 온라인 수업에서 학생들이 느끼는 고립감과 같은 단점을 극복하며, 공간 이동성을 통해 학습공간에서의 공존성을 높인다[9]. 학생들은 메타버스 기반 수업에 대해 사용 편의성, 상호작용성, 몰입감, 흥미성, 실재감을 높게 인식하고 있다[10]. 그리고 비대면 공간 속에서도 많은 사람이 모일 수 있으며 아바타와 같은 대리인을 통해 평등한 위치에서 소통하고 협력하는 기회를 긍정적으로 인식한다. 그리고 수업 참여를 게임처럼 느끼며 재미와 흥미를 느낀다. 그러나 일부 대학생들은 메타버스 공간에서 다른 학습자의 방해, 과도한 표현은 오히려 불편하게 한다고 인식하였다[10]. 또 다른 선행연구[11]에서는 대학생들의 메타버스 기반 교육에 대한 기대와 가치는 높으나 사용 후에는 불만족스러워하는 것으로 보고하였다. 이처럼 현재까지 메타버스를 활용한 교육의 효과는 일관적이지 않게 보고되고 있다. 그러므로 메타버스를 교과목 전반에 활용하기 보다는 전통적인 대면수업과 메타버스를 활용하는 수업을 혼합하는 블랜디드러닝 수업이 적합하다. 또한 수요자인 학생의 관점에서 메타버스 기반 교육에 대한 수용의도와 수용의도에 영향을 미치는 요인을 확인하는 연구가 필요하다[12].

새로운 기술이 도입되면 일부 사용자는 이를 거부할 수 있고, 또 다른 사용자들은 새로운 기술로 인한 변화 자체를 위협으로 느낄 수 있다[13]. 이러한 점에서 대학 교육에 메타버스와 같은 새로운 기술을 도입하고자 할 때는 사용자의 관점에서 기술에 대한 수용의도를 이해하는 것이 필요하다. 새로운 기술에 대한 사용자의 기술수용의도를 설명한 대표적인 이론은 기술수용모델(technology acceptance model, TAM)이다[14]. 이 모델에서 수용의도는 스스로가 이 정보기술을 쉽게 다를 수 있을 것이라는 믿음인 인지된 용이성(perceived ease of use)과 정보기술을 수용하는 것이 개인의 성과를 향상시킬 것이라는 믿음인 인지된 유용성(perceived usefulness)이라는 두 가지 변수에 의해 설명하였다. TAM 2는 TAM에서 제시한 인지된 유용성과 인지된 용이성 외에 주관적 규범(subjective norm), 이미지(image), 업무관련성(job relevance), 결과품질(output quality), 결과입증 가능성(result demonstrability)을 추가하였다[15]. 현재 TAM 2는 다양한 분야에서 새로운 기술을 사용하고자 하는 사용자의 수용의도를 확인할 때 활용되고 있다[16]. 메타버스 기반 교육에 대한 수용의도를 조사한 연구[17]에서 메타버스 기반 교육을 경험한 학생은 경험하지 못한 학생들보다 메타버스의 상호작용성과 즐거움을 더 높게 평가하고, 메타버스 사용의도가 높다고 보고하였다. 또한 대학생은 메타버스를 통해 정보를 획득하고, 상호작용을 하며, 자신을 표현할 수 있고, 메타버스 기반 교육이 자신에게 유용하다고 인지하고 즐거움을 느끼게 되면 메타버스 기반 교육에 대한 이용 만족도가 높아지고 재사용 의도가 높아진다[18]고 보고하였다.

현재 간호교육에서 메타버스는 핵심간호술, 응급간호, 욕창간호, 조현병 환자 간호, 여성시뮬레이션 등 실습 교육의 사전 학습에 활용하고 있었다[19-22]. 이들 선행연구에서는 메타버스를 활용한 교육의 그 효과를 보고하고 있다. 그러나 메타버스 기반 교육을 간호해부학에 적용한 연구는 매우 부족하다. 이에 간호해부학에 메타버스 기반 교육을 도입하기 위해서는 TAM 2를 활용하여 간호대학생들의 메타버스 기반 간호학해부학 수업에 대한 수용의도와 수용의도에 영향을 미치는 요인을 규명하는 연구가 우선적으로 필요하다.

이에 본 연구에서는 한국 간호대학생을 대상으로 TAM 2를 중심으로 메타버스 기반 블랜디드러닝 간호해부학 수업에 대한 수용의도와 수용의도에 영향을 미치는 요인을 규명하고 이를 간호교육에 메타버스를 적용하고자 할 때 기초자료로 제공하고자 한다.

### 2. 연구의 목적

본 연구는 TAM 2를 중심으로 한국 간호대학생의 메타버스 기반 블랜디드러닝 간호해부학 수업에 대한 수용의도와 수용의도에 영향을 미치는 요인을 확인하여, 간호교육에 메타버스를 도입할 때 활용할 수 있는 기초자료로 제시하는 것이며, 구체적인 목적은 다음과 같다.

1) 대상자의 일반적 및 메타버스 관련 특성, TAM 2 변수(주관적 규범, 이미지, 업무관련성, 결과품질, 결과입증가능성, 인지된 용이성, 인지된 유용성)와 수용의도의 정도를 파악한다.

2) 대상자의 일반적 및 메타버스 관련 특성에 따른 수용의도의 차이를 파악한다.

TAM 2 변수(주관적 규범, 이미지, 업무관련성 결과품질, 결과입증가능성, 인지된 용이성, 인지된 유용성)와 수용의도 간의 상관관계를 파악한다.

3) 한국 간호대학생의 메타버스 기반 블랜디드러닝 간호해부학 수업에 대한 수용의도에 영향을 미치는 요인을 파악한다.

## 연구 방법

### 1. 연구 설계

본 연구는 TAM 2를 중심으로 간호대학생의 메타버스 기반 블랜디드러닝 간호해부학 수업 수용의도에 영향을 미치는 요인을 파악하기 위한 서술적 상관관계 연구이다.

### 2. 연구 대상

본 연구는 서울 소재 삼육대학교에서 정규교과 과정으로 메타버스 기반 블랜디드러닝 간호해부학 수업에 참여하였으며 연구의 목적을 이해하고 연구에 참여하는 것을 자발적으로 동의한 간호대학생을 대상으로 하였다. 표본을 수집한 대학은 간호해부학 수업을 메타버스 기반 블랜디드러닝으로 운영하고 있다. 그러나 현재 2학년은 coronavirus disease 2019 (COVID-19) 팬데믹의 종식으로 전면 대면강의 실시라는 교육 정책의 변화로 정규 교육과정에서 메타버스 기반 블랜디드러닝 간호해부학 수업을 수강한 경험이 없어 연구 대상에서 제외하였다.

연구대상자 수를 산출하기 위해 G*Power 3.1.9.7 software (Heinrich-Heine-Universitä Düseldorf, Germany)를 사용하였으며 Lee와 Hong [23]의 연구를 토대로 회귀분석을 위해 유의수준 .05, 검정력 .90, 효과크기 .15, 예측변인 10개를 투입한 결과 산출된 표본의 크기는 147명이었다. 10% 탈락률을 고려하여 165명의 대상으로 설문지를 배부하였고 불성실한 응답을 제외하고 155명을 최종 분석에 사용하였다.

### 3. 연구 도구

#### 1) 일반적 및 메타버스 관련 특성

본 연구에서 연구대상자의 일반적 특성은 3개 문항(성별, 연령, 학년)이며, 메타버스 관련 특성은 5개 문항(간호학 관련 메타버스 교육경험 기간, 간호학 이외 교육에서 메타버스 경험, 메타버스 기반 간호교육 필요성, 메타버스 기반 교육 관심도, 컴퓨터 활용역량)으로 구성하였다. 메타버스 기반 특성 중 메타버스 기반 간호교육의 필요성, 교육 관심도, 컴퓨터 활용역량은 각 1개 문항 5점 척도로 측정하였고 점수가 높을수록 변수에 대한 인식 정도가 높음을 의미한다.

#### 2) TAM 2 변수

본 연구에서 TAM 2 변수는 Venkatesh와 Davis [15]가 개발한 TAM 2 변수 측정 도구를 개발자에게 메일로 사용 승인을 받은 후에 번역하여 사용하였다. 본 연구에서 TAM 2 변수 측정 도구는 영어와 한국어가 가능한 간호학과 교수 1인이 번역하고 연구자와 간호학과 교수 1인이 번역의 정확성과 연구 상황에 필요한 수정 문항이 있는지를 검토하고 수정하였다. 이를 영어와 한국어가 가능한 간호학과 교수 1인이 역 번역하였으며 연구자와 간호학과 교수 1인이 번역의 정확성과 내용의 이해를 재확인하여 도구의 항목을 확정하였다. 이 도구는 8개 영역, 25개 문항 7점 Likert 척도로 개발되었으나 한글로 번역하는 과정에서 개발자의 제안에 의해 5점 척도로 수정하였다. 도구의 내용타당도는 TAM 2 변수를 이용하여 연구한 경험이 있는 간호학과 교수 2인과 경영학과 교수 3인에게 검증받았다.

도구의 내용타당도는 문항 수준의 내용타당도(item-level content validity, I-CVI)와 척도 수준의 타당도(scale-level content validity, S-CVI)로 검증하였다. 특히 S-CVI는 모든 문항 I-CVI 점수 평균(S-CVI index based on the average method, S-CVI/Ave)과 모든 응답자 동의 문항(S-CVI index based on the universal agreement method, S-CVI/UA)으로 검증하였다. I-CVI는 전문가 중 3점 또는 4점으로 응답한 전문가 수를 전체 전문가 수로 나눈 값으로 산출하였다. S-CVI/Ave는 I-CVI 점수의 합계를 전체 문항 수로 나누어서 산출하였으며(S-CVI/Ave = I-CVI 점수의 합계/전체 문항 수) S-CVI/UA는 모든 응답자가 동의한 문항 수를 전체 문항 수로 나누어 산출하였다(S-CVI/UA = 모든 응답의 동의 문항 수/전체 문항 수). S-CVI/UA 점수는 전문가 5명이 모두 3점 또는 4점으로 응답했으면 UA=1이 되고, 5명 중 단 1명이라도 1점 또는 2점으로 응답했으면, UA = 0으로 환산하였으며, S-CVI/UA는 각 문항의 UA에 대한 척도 평균 값을 의미한다. I-CVI 범위는 .80∼1.00, S-CVI/Ave는 .97, S-CVI/UA는 1.00이었다.

본 연구에서 TAM 2 변수는 도구의 하위영역 중 사용의도 2개 문항을 제외하고 7개 영역, 총 23개 문항, 5점 Likert 척도로 수정하여 측정하였다. 본 연구에서 문항별 점수는 ‘매우 그렇다’ 5점부터 ‘매우 그렇지 않다’ 1점으로, 점수가 높을수록 각 영역의 의미가 높음을 의미한다.

도구를 개발한 Venkatesh와 Davis [15]의 연구에서 도구의 신뢰도는 시점별로 제시하였으며 하위영역별 Cronbach’s α는 주관적 규범 .81∼.94, 자발성 .82∼.91, 이미지 .80∼.93, 업무관련성 .80∼.95, 결과품질 .82∼.98, 결과입증가능성 .90∼.97, 인지된 용이성 .86∼.98, 인지된 유용성 .87∼.98이었다. 본 연구에서 Cronbach’s α는 주관적 규범 .90, 이미지 .90, 업무관련성 .89, 결과품질 .82, 결과입증가능성 .87, 인지된 용이성 .83, 인지된 유용성 .94이었다.

#### 3) 수용의도

본 연구에서 수용의도는 Venkatesh와 Davis [15]가 개발한 TAM 2 변수 측정 도구의 하위영역 중 사용의도 2개 문항으로 측정하였다. 본 연구에서 종속변수를 수용의도로 명명한 것은 TAM 2를 적용한 선행연구에서 연구의 목적에 따라 종속변수를 수용의도[24,25], 사용의도[26], 재사용의도[27], 구매의도[28] 등으로 사용함을 근거로 하였다. 본 연구의 목적은 TAM 2를 적용하여 간호대학생의 메타버스 기반 간호해부학수업을 수용의도에 영향을 미치는 요인을 규명하는 것이므로 TAM 2 변수에서 사용의도 2개 문항으로 종속변수인 수용의도를 측정하였다. 본 연구에서 문항별 점수는 ‘매우 그렇다’ 5점부터 ‘매우 그렇지 않다’ 1점으로, 점수가 높을수록 각 영역의 의미가 높음을 의미한다.

도구는 도구 개발자에게 메일로 도구 사용 승인을 받은 후 본 연구에서 번역하여 사용하였다. Venkatesh와 Davis [15]의 연구에서 사용의도의 Cronbach’s α는 .82∼.87이었다. 본 연구에서 사용의도의 Cronbach’s α는 .92이었다.

### 4. 자료 수집

본 연구의 자료 수집은 2023년 11월 1일부터 11월 30일까지 실시하였다. 자료를 수집하기 위해 먼저 연구자는 자료수집 대상 학과의 학과장에게 본 연구의 목적과 취지를 설명하고 자료수집에 대한 승인을 받았다. 이후 학년별 대표들에게 연구목적과 절차를 설명하고 학생들에게 동의를 받은 후에 학생들의 소셜 네트워크 서비스(social networking service, SNS) 단체 대화방에 연구설문지 링크를 공지하였다. 학생들은 연구설문지의 링크를 통해 연구 목적, 방법, 소요 시간이 적힌 설명문을 읽고 연구참여 동의여부를 결정하였다. 자발적으로 연구참여에 동의한 학생들은 메타버스 기반 간호해부학 블랜디드러닝 수업 수용의도라는 온라인 설문지에 응답하도록 하였다.

### 5. 자료 분석

본 연구에서 수집된 자료는 SPSS version 24.0 (IBM Corp., Armonk, NY, USA)으로 분석하였으며 구체적인 방법은 다음과 같다.

1) 일반적 및 메타버스 관련 특성은 빈도와 백분율, 평균과 표준편차를 산출하였다.

2) TAM 2 변수인 주관적 규범, 이미지, 업무관련성, 결과품질, 결과입증가능성, 인지된 용이성, 인지된 유용성, 수용의도는 평균과 표준편차, 왜도 첨도, 신뢰도를 산출하였다.

3) 일반적 및 메타버스 관련 특성에 따른 수용의도의 차이는 독립표본 T 검정(independent samples t-test), 일원분산분석(one-way analysis of variance)로 분석하고, 사후검정은 Scheffé test로 분석하였다.

4) TAM 2 변수인 주관적 규범, 이미지, 업무관련성, 결과품질, 결과입증가능성, 인지된 용이성, 인지된 유용성과 수용의도간의 상관관계는 Pearson correlation coefficient로 분석하였다.

5) 메타버스 기반 블랜디드러닝 간호해부학 수업 수용의도에 영향을 미치는 요인은 위계적 회귀분석(hierarchical multiple regression)으로 분석하였다.

### 6. 윤리적 고려

삼육대학교 기관연구윤리심의위원회 (IRB NO. SYU 2023-09-006) 승인을 받은 후 진행하였다. 연구 참여 동의자가 설문을 위해 링크에 접속하면 첫 화면에서 다시 한번 연구목적, 설문자료의 익명성, 설문중단의 가능성과 이에 대한 불이익이 없음 등의 연구윤리에 대한 부분을 안내하였다. 본 설문 전에 연구설명문을 읽고 동의서에 동의 여부를 표시하도록 하였다. 본 연구는 온라인 설문지로 진행하였기 때문에 연구와 교육을 철저히 구분하여 수업에 지장을 받지 않도록 연구를 진행하는 점과 연구참여자의 학점 부여 등에 영향을 미치지 않음을 명확하게 안내하였다. 수집된 자료는 연구의 목적으로만 사용될 것이며 코딩자료는 암호화되며 연구 종료 시 3년간 보관되며 안전하기 폐기될 것이다. 응답은 약 20분이 소요되었고 참여 대상자에게는 소정의 사례를 제공하였다.

## 연구 결과

### 1. 대상자의 일반적 및 메타버스 관련 특성에 따른 수용의도의 차이

대상자의 성별은 여성이 125명(80.6%)으로, 연령은 21∼23세가 68명(43.9%)으로, 학년은 3학년이 65명(41.9%)으로 가장 많았다. 간호교육에서 메타버스를 활용한 교육 경험은 1학기가 97명(62.6%)으로, 메타버스 기반 교육경험은 있다가 84명(54.2%)으로, 간호교육에서 메타버스 기반 교육의 필요성에 대해서는 필요있다가 66명(42.6%)으로 가장 많았다. 메타버스 기반 교육관심도는 중간 정도가 85명(54.9%)으로 가장 많았고, 본인의 컴퓨터 활용역량은 중간 정도가 110명(71.0%)으로 가장 많았다(Table 1).

대상자 일반적 특성에 따라 수용의도가 통계적으로 유의한 차이가 있는 변수는 연령(F = 5.25, *p* = .006), 학년(F = 10.31, *p* < .001), 간호교육에서 메타버스 기반 교육 필요성 인식(F = 50.21, *p* < .001), 메타버스 기반 교육 관심도(F = 35.20, *p* < .001), 컴퓨터 활용역량(F = 6.05, *p* = .003)이었다. 사후 분석 결과, 수용의도는 21∼23세가 가장 높았으며 학년은 4학년이 가장 높았다. 간호교육에서 메타버스 기반 교육의 필요성에서 필요있다로 응답한 대상자의 수용의도가 가장 높았다. 컴퓨터 활용역량은 상으로 응답한 대상자가 하로 응답한 대상자보다 수용의도가 높았다. 메타버스 기반 교육 관심도는 상으로 응답한 대상자의 수용의도가 가장 높았다. 그러나 성별(t = −1.47, *p* = .143), 간호학에서 메타버스 교육 경험 기간(t = 3.06, *p* = .050), 간호학 이외 교육에서 메타버스 경험(t = 0.16, *p* = .875)에 따른 수용의도는 유의한 차이가 없었다(Table 1).

### 2. TAM 2 변수와 수용의도간의 상관관계

대상자의 TAM 2 변수는 5점 만점으로 조사하였으며, 주관적 규범은 3.16점, 이미지는 2.97점, 업무관련성 3.11점, 결과품질 3.45점, 결과입증가능성 3.58점, 인지된 용이성 3.54점, 인지된 유용성 3.45점이었다. 수용의도는 5점 만점에 3.59점이었다(Table 2). 대상자의 TAM 2 변수와 수용의도간의 상관관계를 확인한 결과, 수용의도와 주관적 규범 (r = .61, *p* < .001), 이미지(r = .59, *p* < .001), 업무관련성(r = .69, *p* < .001), 결과품질(r = .72, *p* < .001), 결과입증가능성(r = .67, *p* < .001) 인지된 용이성(r = .75, *p* < .001) 인지된 유용성(r = .82, *p* < .001)은 모두 유의한 정적 상관관계가 있었다(Table 3).

### 3. 메타버스 기반 블랜디드러닝 간호해부학 수업에 영향을 미치는 요인

연구대상자의 메타버스 기반 블랜디드러닝 간호해부학 수업에 대한 수용의도에 영향을 미치는 요인을 분석하기 위해 2단계 위계적 회귀분석을 실시하였다. 1단계에서는 일반적 특성과 메타버스 관련 특성 중 수용의도와 유의한 차이를 보인 연령, 학년, 메타버스 간호교육 경험, 메타버스 간호교육 필요성, 컴퓨터 활용역량, 메타버스 교육관심을 더미변수로 처리하여 투입하였다. 그리고 2단계에서는 TAM 2 변수 중 수용의도와 유의한 상관관계가 있는 주관적 규범, 인지된 용이성과 인지된 유용성을 추가 투입하였다. 독립변수 간 다중공선성의 유무를 확인하였으며 공차한계(tolerance)는 .20∼.83으로 0.1 이상이었으며, 분산팽창지수(variance inflation factor)는 1.30∼4.99로, 10 이하로 나타나 다중공선성의 문제는 없었다. 오차 잔차의 독립성을 Durbin-Watson으로 검증한 결과, 2.06으로 2에 가깝게 나타나 잔차의 독립성이 검증되어 회귀분석을 위한 가정을 만족하였다.

일반적 특성과 메타버스 관련 특성 중 수용의도에 영향을 미치는 요인을 확인한 모형 1에서 수용의도에 영향을 미치는 변수는 메타버스 교육의 필요성에서 필요있다(β = .59, *p* < .001)와 간호학에서 메타버스 교육 경험이 3학기 이상(β = −.22, *p* = .010)이었으며 회귀모형1의 설명력은 41.0%이었다.

TAM 2 변수 중 수용의도에 영향을 미치는 요인을 확인한 회귀모형2에서 수용의도에 영향을 미치는 변수는 메타버스 교육의 필요성 중에서 필요있다(β =.26, *p* = .004), 인지된 용이성(β = .33, *p* < .001), 인지된 유용성(β = .48, *p* < .001)이었으며 모형 2의 설명력은 76.0%이었다(Table 4).

## 논의

본 연구는 TAM 2를 중심으로 한국 간호대학생의 메타버스 기반 블랜디드러닝 간호해부학 수업 수용의도 정도와 수용의도에 영향을 미치는 요인을 확인하였으며, 그 결과를 토대로 논의하고자 한다.

본 연구에서 간호대학생의 메타버스 기반 블랜디드러닝 간호해부학 수업에 대한 수용의도는 5점 만점에 3.59점이었다. 이는 같은 도구로 측정한 연구가 없어서 직접 비교는 어렵지만 간호대학생을 대상으로 메타버스 기반 해부학실습에 대한 기술수용도를 조사한 연구[17]에서 수용도를 5점 만점에 4.10점으로 보고한 결과보다 낮았다. 본 연구는 간호해부학 수업에 대한 메타버스의 수용의도를 조사한 반면 선행연구[17]는 해부학실습 교육에 메타버스나 가상현실 기술을 적용한 수업에 대한 수용의도를 조사하였기 때문으로 생각한다. 이는 간호대학생들은 이론적 개념을 전달하는 이론 교육보다는 교수-학생간 상호작용과 반복 연습이 필요한 실습 교육에 혁신적인 교수학습 전략을 활용하는 것이 더 효과적이라고 생각하는 것으로 설명할 수 있다[29]. 실제로 현재 간호교육에서 4차 산업과 관련된 기술인 메타버스[19], VR [6], AR [30]은 주로 간호술기 교육에 적용되고 있다. 그러나 현재 대학에서 교육받는 밀레니엄 Z세대(Millennial Generation Z)세대는 인터넷을 기반으로 스마트폰을 사용하고 다양한 앱과 SNS 활동이 익숙한 디지털 네이티브 세대이다[31]. 또한 COVID-19 팬데믹으로 인해 고등학교 때부터 동영상 강의, 실시간 화상 강의 등을 경험한 세대로 디지털 모바일 매체뿐 아니라 동영상 등 다양한 콘텐츠를 능숙하게 활용하는 세대이다[32]. 그러므로 간호학 전공이론 수업에 메타버스 기반 교수학습 방법을 적용하고자 하는 노력이 필요하다.

그러나 간호대학생을 대상으로 한 본 연구와 선행연구[23,30]의 결과는 교육대학의 예비교사를 대상으로 한 연구[33]에서 메타버스 수용의도를 5점 만점에 2.55점으로 보고한 결과보다 높았다. 이는 메타버스나 가상현실과 같은 새로운 기술에 대한 학생들의 수용의도가 교사들의 수용의도보다 높음을 의미한다. 이는 새로운 기술을 교육에 도입할 때, 학생들은 사용자 입장에서 새로운 기술을 수용하면 된다. 그러나 교사들은 기술을 교육도구로 활용하기 위해 많은 노력이 필요하기 때문에 부담감을 크게 느끼는 것으로 설명할 수 있다. 이는 메타버스를 간호교육에 활용하기 위해서는 교육자들이 새로운 기술을 교육에 효과적으로 활용할 수 있도록 대학에서는 필요한 기술 지원과 함께 기술 인프라를 구축할 것을 제안한다.

본 연구에서 간호대학생의 메타버스 기반 간호해부학 블랜디드러닝 수업 수용의도에 영향을 미치는 요인을 확인하기 위해 위계적 회귀분석을 실시하였다. 회귀모형1에서는 일반적 특성과 메타버스 관련 특성을 투입한 결과, 교육의 필요성과 메타버스 교육 경험이 유의한 요인으로 확인되었다. 이는 간호대학생을 대상으로 TAM 2를 적용하여 수용의도에 영향을 미치는 요인을 규명한 연구가 없어서 직접 비교는 어렵지만, 간호대학생을 대상으로 가상현실 기반 교육프로그램의 사용의도를 조사한 연구[23]에서 가상현실 기반 교육에 대한 기대감, 가상현실 교육에 대한 필요성이 사용의도에 영향을 미치는 것으로 보고한 결과와 유사하였다. 또한 일반 대학생을 대상으로 한 연구[10]에서도 메타버스 유경험자들이 무경험자보다 사용의도가 높은 것으로 보고한 결과와도 일치한다. 이는 간호대학생이 메타버스 기반 교육의 필요성을 높게 인지할수록, 메타버스 기반 교육 경험이 많을수록 메타버스 수용의도가 높아진다는 것을 의미한다. 그러므로 간호해부학 수업에 메타버스를 활용하고자 한다면 먼저 학생들에게 메타버스 기반 교육의 필요성을 인식할 수 있도록 설명하는 것이 필요하다. 그리고 간호해부학 수업 전에 메타버스를 활용할 수 있는 기회를 제공하여 학생들이 메타버스 기반 교육에 익숙해질 수 있도록 하는 전략이 필요하다고 생각한다.

본 연구에서 TAM 2 변수 중에서 간호대학생의 메타버스 기반 블랜디드러닝 간호해부학 수업 수용의도에 영향을 미치는 요인을 확인한 회귀모형2에서 메타버스 기반 교육 경험, 인지된 용이성과 인지된 유용성이 수용의도에 영향을 미치는 요인으로 확인되었으며, 이들 변수는 수용의도를 72% 설명하였다. 이는 메타버스 기반 학습경험이 많을수록, 인지된 용이성과 인지된 유용성이 높을수록 메타버스 기반 블랜디드러닝 간호해부학 수업에 대한 수용의도가 높다는 것을 의미한다. 본 연구와 동일한 도구를 활용한 연구가 아니라 직접 비교는 어렵지만, 간호대학생과 일반 학생을 대상으로 한 연구[15,18]에서 인지된 용이성과 인지된 유용성을 수용의도에 중요한 영향 요인으로 보고한 결과와 일치한다. 또한 TAM을 중심으로 메타버스 기반 교육의 수용의도를 검증한 연구[14]에서 메타버스에 대해 인지된 유용성과 인지된 용이성을 높게 지각할수록 메타버스 이용에 대한 태도가 긍정적이었으며, 메타버스에 대한 긍정적 태도는 메타버스의 이용의도를 높였다고 보고한 결과와도 유사하였다. 그러나 AR을 활용한 해부학 수업에 참여한 간호대학생을 대상으로 AR을 활용한 학습방법 사용성(usability)에 인지된 유용성이 영향을 미치며 설명력은 51.2%로 보고한 연구[30]와 VR을 활용한 수업 수용성에 인지된 유용성과 인지된 즐거움이 유의미한 영향을 미쳤으며, 설명력을 70.9%로 보고한 결과[34]와는 차이가 있었다. AR과 VR은 주로 개별적 체험을 제공하는 몰입형 기술로서 학습자가 특정 환경이나 대상을 실감나게 경험하도록 돕는 도구적 성격이 강하기 때문에 인지된 유용성이 영향을 미친 것[35]으로 판단된다. 또한 AR과 VR 기술은 이미 다양한 분야에서 활용되고 있어 인지된 용이성보다는 인지된 유용성이 수용의도에 영향을 미친 것으로 생각한다. 반면 메타버스는 AR과 VR 기술들을 포괄하면서도 학습자와 교수자뿐 아니라 학습자 간의 상호작용, 정체성 표현, 공동체 소속감을 함께 수용하게 한다는 점에서 차별화되는 플랫폼이다. 그리고 메타버스는 최근 교육에 활용되기 시작하였기 때문에 인지된 유용성과 인지된 용이성이 수용의도에 영향을 미친 것으로 설명할 수 있다[36]. 그러므로 추후 학생들의 메타버스를 활용한 교육에 대한 수용의도를 높이기 위해 기술적 수용성뿐 아니라 사회적 상호작용성, 정체성 표현과 공동체 참여의식과 관련된 경로를 추가하여 검증하는 것이 필요하다[37].

본 연구에서 TMA 2를 중심으로 간호대학생의 메타버스 기반 블랜디드러닝 간호해부학 수업에 대한 수용의도를 설명하는 회귀모형의 설명력은 72%로 높은 편이었다. 이는 비록 다중공선선 검증에서 통계적으로 문제가 없다고 판단되었지만 인지된 유용성과 인지된 용이성 간에 상관관계가 확인되었고, 1개 대학에서 표본을 추출하였다는 점에서 과적합(overfitting) 가능성을 완전히 배제하기는 어렵다. 따라서 향후 연구에서는 교차타당화(cross-validation) 기법을 적용하거나, 다양한 대학과 집단을 대상으로 한 재검증을 통해 모형의 일반화 가능성을 확보할 필요가 있다. 본 연구 결과를 근거로 간호대학생의 메타버스를 활용한 간호해부학 수업에 대한 수용의도를 높이기 위해서는 메타버스를 개발할 때 학생들이 활용하기 쉽게 설계해야 할 것이다. 또한 암기해야 할 내용이 많은 어려운 교과목이 아니라 재미있으며 향후 전공교과목의 기초를 학습한다는 동기를 가질 수 있도록 메타버스 기반 수업에서 퍼즐, 방탈출 게임, OX 퀴즈, 전공교과목과의 연계성을 찾아갈 수 있도록 개발하는 전략이 필요하다. 그러나 간호대학생을 대상으로 AR [30]과 VR [34]을 간호교육에 활용하고자 할 때 인지된 유용성만 수용의도에 영향을 미치는 요인으로 보고한 결과는 본 연구와 차이가 있다. 이와 같은 차이는 선행연구[30,34]에서 수용의도를 조사한 본 연구는 4차 산업과 관련된 기술 중 메타버스 기반 간호학 교육을 경험한 간호대학생을 대상으로 하였으나 선행연구[30,34]는 VR이나 AR을 실습 교육에 적용하는 것에 대한 수용의도와 관련된 요인을 규명한 연구이기 때문으로 판단된다. 그러나 본 연구와 선행연구에 참여한 학생들은 COVID-19 팬데믹 때문에 다양한 매체를 통한 온라인 학습을 경험하였기 때문에 인지된 유용성이 수용의도에 영향을 미친 것으로 판단된다. 그러나 예비교사의 메타버스 기반 교육에 대한 수용의도에는 인지된 용이성만이 메타버스 수용의도에 유의한 영향을 미쳤다고 보고한 결과[33]와는 차이가 있었다. 이는 메타버스를 교육에 활용하기 위해서는 학습자인 간호대학생의 인지된 유용성을 높이기 위한 전략도 필요하지만, 교육자의 메타버스 활용 능력을 높일 수 있는 전략도 필요함을 의미한다.

본 연구는 1개 대학에서 간호대학생을 편의표집하였으며, 간호해부학 수업에 대한 메타버스 기반 블랜디드러닝에 대한 수용의도를 조사하였기 때문에 연구 결과를 전체 간호대학생과 간호학 전공교과목에 일반화하는데 한계가 있다. 그러나 본 연구는 간호교육에 메타버스를 활용하기 위한 교육전략을 수립할 때 중요한 근거를 제공하였다는데 의의가 있다. 본 연구는 TAM 2의 모델을 중심으로 변수를 선정하고 이들 변수가 수용의도에 미치는 영향을 연구하였다. 그러나 추후에는 선행연구를 토대로 의미 있게 검증된 변수로 독립변수를 확대하여 변수 간의 관계를 구조모형으로 검증할 필요가 있다. 또한 간호대학생의 메타버스 기반 교육에 대한 수용의도를 높일 수 있는 전략을 적용한 교육프로그램을 개발하고 그 효과를 확인하는 중재연구도 필요하다.

## 결론

본 연구는 TAM 2를 중심으로 한국 간호대학생의 메타버스 기반 블랜디드러닝 간호해부학 수업 수용의도에 영향을 미치는 요인을 파악하기 위한 서술적 조사 연구이다. 본 연구에서 간호대학생의 메타버스 기반 간호해부학 블랜디드러닝 수업 수용의도에 영향을 미치는 요인은 교육의 필요성, 인지된 유용성, 인지된 용이성으로 확인되었다. 이를 근거로 메타버스를 간호해부학 수업에 활용하기 위해서는 간호대학생들이 메타버스 기반 블랜디드러닝 간호해부학 수업의 필요성뿐 아니라 유용성에 대해 인식할 수 있는 설명과 함께 체험할 수 있는 기회를 제공하는 것이 필요하다. 또한 메타버스 체계를 구축할 때 이용자가 쉽게 사용할 수 있도록 설계하고, 활용 방법에 대한 지침이나 메뉴얼을 제공해야 할 것을 제안한다. 그리고 간호대학생들의 메타버스 기반 교육에 대한 인지된 유용성을 높이기 위해 학생의 사전교육요구도를 파악하여 교육내용을 설계할 것을 제안한다.

## Figures and Tables

**Table 1.** Differences in Intention to Use According to Participants’ Characteristics (N = 155)

Characteristics	Categories	n (%)	Intention to use		
			M ± SD	t/F	*p* (Scheffe)
Sex	Men	30 (19.4)	3.37 ± 0.96	−1.47	.143
	Women	125 (80.6)	3.64 ± 0.92		
Age (years)	18∼20^a^	55 (35.5)	3.27 ± 0.91	5.25	.006 (a < b)
	21∼23^b^	68 (43.9)	3.77 ± 0.84		
	≥ 24^c^	32 (20.6)	3.75 ± 1.03		
Year in program	1st^a^	63 (40.7)	3.27 ± 0.94	10.31	< .001 (a,b < c)
	3rd^b^	65 (41.9)	3.64 ± 0.94		
	4th^c^	27 (17.4)	4.18 ± 0.44		
Duration of metaverse-based education experience in nursing (semester)	1	97 (62.6)	3.52 ± 0.99	3.06	.050
	2	33 (21.3)	3.94 ± 0.79		
	≥3	25 (16.1)	3.40 ± 0.74		
Experience with metaverse-based education outside nursing	Yes	84 (54.2)	3.60 ± 0.97	0.16	.875
	No	71 (45.8)	3.58 ± 0.89		
Need for metaverse nursing education	No^a^	25 (16.1)	2.66 ± 0.98	50.21	< .001 (a < b < c)
	Neutral^b^	64 (41.3)	3.30 ± 0.76		
	Yes^c^	66 (42.6)	3.60 ± 0.93		
Interest in the metaverse program	Low^a^	34 (21.9)	2.79 ± 0.84	35.20	< .001 (a < b < c)
	Middle^b^	85 (54.9)	3.59 ± 0.80		
	High^c^	36 (23.2)	4.35 ± 0.62		
Ability to use computers	Low^a^	23 (14.8)	3.00 ± 0.98	6.05	.003 (a < c)
	Middle^b^	110 (71.0)	3.67 ± 0.84		
	High^c^	22 (14.2)	3.82 ± 0.93		

M = Mean; SD = Standard deviation.

**Table 2.** Degree of Technology Acceptance Model 2 Variables (N = 155)

Variables	Range	M ± SD	Skew	Kurt	Cronbach’s α
Subjective norm	1∼5	3.16 ± 0.93	−0.09	0.43	.90
Image	1∼5	2.97 ± 0.96	0.20	−0.07	.90
Job relevance	1∼5	3.11 ± 0.97	−0.19	−0.19	.89
Output quality	1∼5	3.45 ± 0.90	−0.50	0.33	.83
Result demonstrability	1∼5	3.58 ± 0.72	−0.59	1.39	.88
Perceived ease of use	1∼5	3.54 ± 0.81	−0.03	−0.22	.83
Perceived usefulness	1∼5	3.45 ± 0.95	−0.28	−0.39	.95
Intention to use	1∼5	3.59 ± 0.93	−0.43	−0.09	.92

M = Mean; SD = Standard deviation.

**Table 3.** Correlations among Technology Acceptance Model Variables (N =155)

Variables	Intention to use
	r (*p*)
Subjective norm	.61 (< .001)
Image	.59 (< .001)
Job relevance	.69 (< .001)
Output quality	.72 (< .001)
Result demonstrability	.67 (< .001)
Perceived ease of use	.75 (< .001)
Perceived usefulness	.82 (< .001)

**Table 4.** Factors Influencing Nursing Students' Intention to Accept Metaverse-based Blended Learning Nursing Anatomy Classes (N = 155)

Independent variables		Model 1					Model 2				
		B	SE	β	t	*p*	B	SE	β	t	*p*
(Constant)		2.59	.20		13.22	< .001	0.26	.23		1.13	.259
Age (years)	18∼20 (ref.)										
	21∼23	0.04	.24	.02	0.17	.868	0.06	.17	.03	0.36	.717
	≥ 24	−0.24	.31	−.09	−0.79	.432	−0.14	.21	−.05	−0.68	.499
Year in program	1st (ref.)										
	3rd	1.07	.81	.10	1.31	.192	0.57	.54	.05	1.04	.300
	4st	0.46	.27	.24	1.72	.087	−0.03	.19	−.02	−0.18	.856
Need for metaverse nursing education	No (ref.)										
	Neutral	0.33	.22	.17	1.51	.134	0.20	.14	.10	1.42	.159
	Yes	1.17	.26	.59	4.44	< .001	0.52	.18	.26	2.91	.004
Duration of the metaverse nursing education (semester)	1 (ref.)										
	2	0.01	.21	.00	0.04	.972	0.12	.14	.05	0.90	.368
	≥ 3	−0.59	.23	−.22	−2.60	.010	0.02	.15	.01	0.12	.902
Ability to use computers	Low (ref.)										
	Middle	0.02	.20	.01	0.09	.926	−0.07	.13	−.04	−0.57	.569
	High	−0.09	.27	−.02	−0.26	.798	−0.19	.17	−.07	−1.12	.267
Interest in the metaverse program	Low (ref.)										
	Middle	0.16	.20	.08	0.79	.429	−0.07	.13	−.04	−0.51	.615
	High	0.40	.29	.17	1.40	.165	−0.20	.19	−.08	−1.03	.306
Subjective norm							0.05	.06	.05	0.79	.429
Perceived ease of use							0.38	.09	.33	4.34	< .001
Perceived usefulness							0.49	.07	.48	6.69	< .001
F (*p*)		8.34 (< .001)					27.10 (< .001)				
R^2^		.47					.78				
Adjusted R^2^		.41					.76				
R^2^ (adjusted R^2^) Change							.31 (.35)				

SE = Standard error; ref. = Reference.

## Data Availability

The data that support the findings of this study are available from the corresponding author upon reasonable request.
